# Plastic film mulching enhances flavonoid accumulation in the roots of *Scutellaria baicalensis*

**DOI:** 10.3389/fgene.2026.1739808

**Published:** 2026-02-18

**Authors:** Bin Ma, Ying Niu, Yang-Mei Bao, Hua Liu, Dongqing Wang, Wenjin Zhang, Ming Li, Lan-Ping Guo

**Affiliations:** 1 Institute of Forestry and Grassland Ecology, Ningxia Academy of Agricultural and Forestry Sciences, Yinchuan, China; 2 Key Laboratory of Desertification Control and Soil and Water Conservation of Ningxia, Yinchuan, China; 3 State Key Laboratory for Quality Ensurance and Sustainable Use of Dao-di Herbs, National Resource Center for Chinese Materia Medica, China Academy of Chinese Medical Sciences, Beijing, China; 4 School of Pharmacy, Ningxia Medical University, Yinchuan, China; 5 Ningxia Specialized Engineering Research Center for Modernization of Traditional Chinese Medicine / Liupan Mountain Regional Medicinal Resources Protection and Development Key Laboratory of the Ministry of Education / Ningxia Key Laboratory for the Modernization of Ethnic Minority Medicine, Yinchuan, China

**Keywords:** flavonoid biosynthesis, multi-omics, plastic film mulching, Scutellaria baicalensis, WRKY

## Abstract

**Background:**

*Scutellaria baicalensis* Georgi is a medicinal herb of considerable importance, valued for its roots that are enriched with flavonoids underlying its pharmacological properties. A principal challenge in its commercial cultivation is the simultaneous enhancement of root biomass and the concentration of these active constituents. Although plastic film mulching is a well-established agronomic practice, its application to *S. baicalensis* and the corresponding molecular regulatory mechanisms remain insufficiently characterized.

**Methods:**

This study systematically evaluated the effects of single- and double-layer plastic film mulching relative to an unmulched control on the agronomic traits of *S. baicalensis*. We employed an integrated analytical strategy, combining transcriptomic and broadly targeted metabolomic analyses of root and leaf tissues. Key regulatory networks and metabolic pathways were subsequently elucidated through weighted gene co-expression network analysis and pathway enrichment.

**Results:**

Film mulching significantly promoted plant growth, with the double-layer application increasing root biomass by 417% compared to the control. Multi-omic analyses revealed a profound reprogramming of root transcription and metabolism in response to mulching. Transcriptomic shifts indicated the activation of central carbon metabolism, providing the energetic foundation for biomass accretion. Concurrently, metabolomic profiles confirmed a substantial increase in the accumulation of core flavonoids, including baicalin and baicalein. Integrative analysis implicated a regulatory network governed by WRKY and AP2/ERF transcription factors as central to this response. The upregulation of these transcription factors coincided with the synergistic activation of downstream enzyme genes in the flavonoid biosynthesis pathway, culminating in elevated flavonoid content.

**Conclusion:**

Our findings demonstrate that plastic film mulching optimizes the rhizosphere to not only activate the primary metabolism requisite for rapid growth but also, acting as an environmental cue, to initiate a WRKY- and AP2/ERF-centric regulatory cascade that systematically enhances root-specific flavonoid biosynthesis. This work provides a molecular rationale for employing this agronomic strategy to achieve high-yield, high-quality cultivation of *S. baicalensis*.

## 1 Introduction

*Scutellaria baicalensis* Georgi, a perennial herb of the Lamiaceae family, is a cornerstone of traditional Chinese medicine, with a medicinal history of its dried roots spanning over a millennium (Zhao et al., 2019). Modern phytochemical analyses have attributed its pharmacological activities to a unique profile of flavonoids enriched in the roots, among which baicalin, baicalein, and wogonin are considered the core active constituents (Li-Weber, 2009; Zhao et al., 2021). Consequently, the ultimate medicinal and economic value of *S. baicalensis* is directly determined by the dual criteria of root biomass accumulation and the concentration of these key chemical components. In commercial cultivation practices, however, a pivotal challenge arises from the pronounced sensitivity of its yield and chemical quality to environmental stressors, particularly drought and temperature fluctuations; this instability severely impedes the standardization process for its medicinal materials (Cheng et al., 2018; Yuan et al., 2011). The development of agronomic strategies that can concurrently stabilize and enhance both yield and quality is therefore of significant importance for the *S. baicalensis* industry.

As a potent agronomic intervention, plastic film mulching is founded on its capacity to engineer the physical properties of the soil microenvironment, a practice that has been validated globally as a reliable means to enhance the productivity and resource-use efficiency of diverse crops (Gao et al., 2019). The primary mechanism of film mulching involves its direct modulation of the soil surface energy balance; by altering the surface albedo and emissivity, it increases the absorption of short-wave solar radiation while reducing long-wave radiation loss, thereby effectively elevating the temperature of the arable soil layer. In temperate regions, this improvement in thermal conditions has been shown to permit earlier sowing and accelerate the early vegetative growth stages of maize (*Zea mays*) (Amare and Desta, 2021). In parallel, film mulching acts as a physical barrier at the hydrological level. By obstructing the pathway for vapor exchange between the soil and the atmosphere, it substantially suppresses surface evaporation, leading to a marked improvement in water-use efficiency. On the Loess Plateau of semi-arid China, for instance, film mulching can maintain relatively high soil moisture content throughout the growing season for wheat (*Triticum aestivum*) and potato (*Solanum tuberosum*), thereby mitigating the constraints of seasonal drought on yield (Yang et al., 2018; Yang et al., 2023). These optimized hydro-thermal conditions, in turn, directly accelerate the rates of cell division and subsequent elongation in the root apical meristems and systemically enhance the root’s capacity for acquiring key mineral nutrients, such as nitrogen and phosphorus, by improving their mobility in the soil solution and potentially accelerating microbial mineralization of organic matter (Wang et al., 2022).

The cascade of microenvironmental alterations induced by film mulching is recognized by the plant’s root sensory system as a complex physiological signal, which subsequently triggers profound metabolic reprogramming within the cells. Research in *Salvia miltiorrhiza*, for example, has demonstrated that film mulching treatments can significantly upregulate the transcription of key genes in the salvianolic acid biosynthesis pathway, such as *SmPAL1* and *SmTAT1*, culminating in a nearly 30% increase in the accumulation of salvianolic acid B in the roots (Pan et al., 2024). Similarly, in the cultivation of *Glycyrrhiza uralensis*, the elevated soil temperatures resulting from mulching were found to be significantly and positively correlated with the content of glycyrrhizic acid and liquiritin, an effect linked to the upregulation of multiple transcription factors regulating terpene and flavonoid synthesis, including *GuWRKY1* (Yang et al., 2022). The mild thermal stress, altered soil gas composition, and stable moisture conditions engendered by mulching can all be perceived by root cell membrane receptors and transduced through intricate signaling cascades, involving, for instance, mitogen-activated protein kinase pathways and phytohormones such as abscisic acid and jasmonic acid, ultimately converging on the activation of specific transcription factors. In cotton (*Gossypium hirsutum*), elevated temperatures under plastic mulch have been confirmed to induce *GhWRKY22* expression, leading to ROS accumulation and premature leaf senescence (Qi et al., 2025). These activated transcription factors subsequently bind to the promoter regions of key enzyme genes in secondary metabolic pathways, thereby systemically increasing the metabolic flux through these pathways and channeling a greater proportion of primary carbon metabolites toward the biosynthesis of specialized compounds such as phenolics and terpenoids (Yang et al., 2018). Thus, plastic film mulching both drives biomass accumulation through the optimization of physical conditions and reshapes the plant’s secondary metabolic network through the introduction of perceivable environmental signals.

To elucidate the mechanisms by which film mulching governs the growth dynamics and metabolic networks of *S. baicalensis*, this study employed an integrated transcriptomic and metabolomic analytical strategy. Despite the widespread use of plastic film mulching, its specific molecular impact on the secondary metabolism of *S. baicalensis*—particularly under different mulching intensities—remains unexplored. We established varying levels of film mulching treatments to comprehensively investigate the responses of *S. baicalensis* across three tiers: agronomic traits, gene expression profiles, and overall metabolite landscapes. This study aims to fill the research gap by identifying the specific pathways and regulatory hubs that respond to mulching signals. We expect that an integrated analysis of the transcriptome and metabolome will reveal a coordinated regulatory network between primary growth and flavonoid biosynthesis. By providing a molecular rationale for biomass and quality enhancement, this work provides a robust theoretical framework for optimizing the standardized cultivation of *S. baicalensis* through agronomic measures and offers new paradigms for dissecting the profound biological principles underlying crop responses to cultivation practices.

## 2 Materials and methods

### 2.1 Experimental design and plant material

The field experiment was conducted in an experimental field located in Yuwang Township, Tongxin County, Wuzhong City, Ningxia, China. Two-year-old seedlings of *Scutellaria baicalensis* Georgi were used as the plant material. Three treatments were established: a control (T1) with conventional field management without mulching; a single-layer mulch treatment (T2) with the application of one layer of standard agricultural plastic film; and a double-layer mulch treatment (T3) with the application of two layers of the same film.

The experiment followed a completely randomized block design with three biological replicates for each treatment, totaling nine plots. Each plot measured 6 m × 6 m and was randomly arranged. Throughout the growing period, all agronomic practices, including irrigation, fertilization, and weed control, were uniformly applied across all plots, with the exception of the mulching treatments.

### 2.2 Phenotypic trait measurement and sample collection

At the harvest stage of *S. baicalensis* on September 2, 2024, five plants exhibiting uniform growth were randomly selected from each plot for agronomic trait measurement. The following parameters were recorded: above-ground height, measured from the soil surface to the plant apex; total plant length, measured from the root tip to the plant apex after excavation; root fresh weight, weighed on an analytical balance after separation from the shoot and thorough washing; and root diameter, measured at the thickest part of the main root using electronic calipers.

Immediately following phenotypic measurements, leaf (TL) and root (TR) samples were collected. This sampling strategy yielded a total of 18 samples, corresponding to the three biological replicates for each of the three treatments and two tissue types. The samples were immediately flash-frozen in liquid nitrogen and stored in an ultra-low temperature freezer at −80 °C for subsequent transcriptomic and metabolomic analyses.

### 2.3 Transcriptome sequencing and analysis

Total RNA was extracted from the 18 samples using a standard RNA extraction kit (DP441, Tiangen Biotech, Beijing, China). The concentration, purity, and integrity of the extracted RNA were assessed using a NanoDrop spectrophotometer and an Agilent 2100 Bioanalyzer. RNA samples with an RNA Integrity Number greater than 8.0 were selected for library construction. Sequencing libraries were prepared using the NEBNext® Ultra™ RNA Library Prep Kit for Illumina® (NEB, Ipswich, MA, United States), and high-throughput sequencing was performed on an Illumina NovaSeq 6000 platform (Biomarker Technologies, Beijing, China). Raw sequencing reads were subjected to quality control by removing adapter sequences and low-quality reads to ensure that the percentage of bases with a quality score above 30 exceeded 94%.

### 2.4 DEG screening and functional enrichment analysis

High-quality clean reads were mapped to the *S. baicalensis* reference genome using HISAT2 software. Gene expression levels were quantified as Fragments Per Kilobase of transcript per Million mapped reads. Differentially expressed genes were identified using the DESeq2 R package with criteria of a fold change ≥2 and a false discovery rate <0.01. Kyoto Encyclopedia of Genes and Genomes pathway enrichment analysis was performed on the differentially expressed genes to identify their primary biological functions. Gene enrichment analyses and visualizations were conducted using the clusterProfiler R package. Venn diagrams and heatmaps were generated using TBtools (v2).

### 2.5 Broadly targeted metabolomics analysis

Samples stored at −80 °C were ground to a fine powder in liquid nitrogen, and the powder was accurately weighed. Metabolites were extracted with 1000 μL of a pre-chilled mixture of methanol, acetonitrile, and water (1:2:1, v/v/v). The mixture was vortexed for 30 s, ground at 45 Hz for 10 min, and sonicated in an ice-water bath for 10 min. After standing at −20 °C for 1 h, the mixture was centrifuged at 12,000 rpm at 4 °C for 15 min. The resulting supernatant was filtered through a 0.22 μm organic membrane for UPLC-MS/MS analysis. An equal aliquot of extract from each sample was pooled to create a quality control sample. Analysis was performed on a UPLC-MS/MS system comprising a Waters Acquity I-Class PLUS UPLC and an Applied Biosystems 6500 Q TRAP mass spectrometer. Chromatographic separation was achieved on a Waters HSS T3 column (1.8 μm, 2.1 mm × 100 mm) with a mobile phase consisting of 0.1% formic acid and 5 mM ammonium acetate in water (A) and acetonitrile with 0.1% formic acid (B). The gradient program was as follows: 0–1.5 min, 2% B; 1.5–5.0 min, 2%–50% B; 5.0–9.0 min, 50%–98% B; 9.0–10.0 min, 98% B; 10.0–11.0 min, 98%–2% B; 11.0–14.0 min, 2% B. The flow rate was 0.35 mL/min, the column temperature was 50 °C, and the injection volume was 4 μL. Mass spectrometry was operated in multiple reaction monitoring mode with the following ESI source parameters: source temperature, 550 °C; ion spray voltage, 5500 V (positive) or −4500 V (negative); ion source gas I, 50 psi; ion source gas II, 55 psi; and curtain gas, 35 psi. The collision-activated dissociation gas was set to medium.

Data acquisition and processing were performed using Analyst software. Metabolites were identified and quantified by comparison against the GB-Plant database and public libraries. The raw peak areas were normalized to the total peak area. Principal component analysis and orthogonal partial least squares-discriminant analysis (OPLS-DA) were employed to assess the overall metabolic differences among the treatment groups. Principal component analysis was conducted using the PCAtools R package.

### 2.6 WGCNA and pathway analysis

To identify key gene modules associated with tissue specificity and responses to the mulching treatments, a weighted gene co-expression network analysis was performed on 16,635 genes exhibiting high expression levels and variance across all samples. A soft-thresholding power of 14 was selected to construct a scale-free network using the WGCNA R package. Gene modules were identified using the dynamic tree cut algorithm with a minimum module size of 30 genes, and modules with a similarity threshold above 0.25 were merged. Pearson correlation coefficients were calculated between the module eigengenes and sample traits. The co-expression network was visualized using Cytoscape.

Differentially expressed genes and differentially expressed metabolites were co-mapped to the KEGG database for an integrated analysis of key pathways, including glycolysis/gluconeogenesis and flavonoid biosynthesis, to reveal the coordinated regulatory mechanisms under film mulching.

### 2.7 qRT-PCR validation of key genes

To validate the RNA-seq data, 20 key genes implicated in the multi-omic analyses, including transcription factors and key enzyme genes, were selected for quantitative real-time PCR analysis. Total RNA from the same batches used for sequencing was reverse-transcribed into cDNA using the PrimeScript™ RT Reagent Kit (TaKaRa, Dalian, China). Quantitative real-time PCR was performed using SYBR® Premix Ex Taq™ II (TaKaRa) on a CFX96 Real-Time PCR Detection System (Bio-Rad, Hercules, CA, United States). The *Actin* gene of *S. baicalensis* served as the internal reference gene for normalization. Relative gene expression levels were calculated using the 2^−ΔΔCt^ method. Bar charts and linear regression analyses were generated using GraphPad Prism. The primer list used in this study are in Supplementary Table S1.

## 3 Result

### 3.1 Plastic film mulching promotes the growth of *S. baicalensis*

**FIGURE 1:**
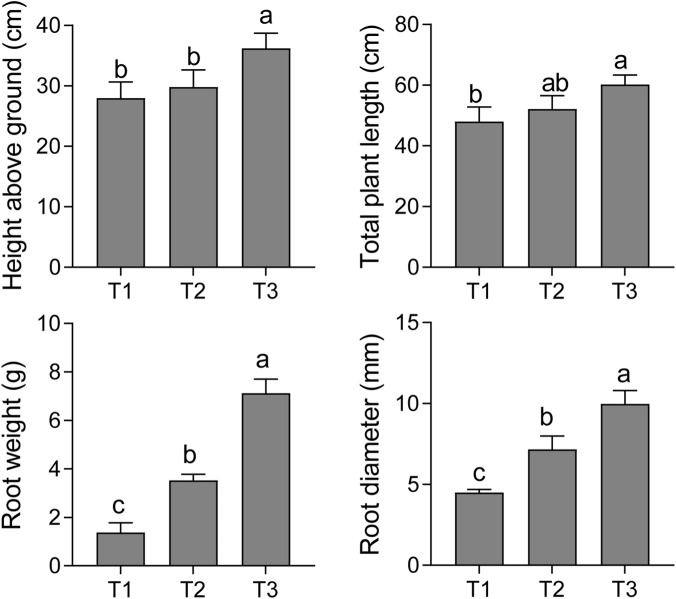
Effects of different plastic film mulching treatments on the phenotypic traits of *S. baicalensis*. The figure displays four key agronomic traits under T1 (unmulched control), T2 (single-layer mulch), and T3 (double-layer mulch) treatments, including height above ground, total plant length, root weight, and root diameter. Data are presented as the mean ± SD. Different lowercase letters (a, b, c) above the bars indicate significant differences between treatment groups at the *p* < 0.05 level, as determined by one-way ANOVA followed by an LSD test.

To systematically evaluate the macroscopic effects of different plastic film mulching strategies on the agronomic traits of *S. baicalensis*, we conducted detailed phenotypic measurements and statistical analyses on plants from the T1 (unmulched control), T2 (single-layer mulch), and T3 (double-layer mulch) treatments (Figure 1). The results unequivocally demonstrated that film mulching, as an effective agronomic practice, significantly promoted the overall growth and development of *S. baicalensis*. Furthermore, this promotional effect exhibited a positive, dose-dependent relationship with the number of mulch layers.

In the aerial parts, both the T2 and T3 treatments resulted in a significant enhancement of plant height compared to the unmulched control (T1). Specifically, the average above-ground height in the T3 treatment group reached 36.2 ± 2.5 cm, representing an approximate 29.3% increase over the 28 ± 2.6 cm observed in the T1 group (*p* < 0.05). Similarly, for the comprehensive trait of total plant length, the T3 treatment also exhibited optimal performance, with an average length of 60.2 ± 3.1 cm, a significant 25.5% increase relative to the T1 control (48.0 ± 4.9 cm). These data indicate that film mulching, particularly with a double layer, effectively promotes the development of photosynthetic organs and vegetative growth in *S. baicalensis* by improving the soil microenvironment.

The growth-promoting effect of film mulching was particularly pronounced on the subterranean root system, a factor of paramount economic importance for *S. baicalensis*, as the root is the primary medicinal part. The average root fresh weight in the T1 control was merely 1.38 ± 0.40 g, whereas in the T2 and T3 treatments, it reached 3.52 ± 0.25 g and 7.13 ± 0.58 g, respectively. Notably, the root weight in the T3 treatment surged by 430% compared to T1, a highly significant difference (*p* < 0.01). This finding highlights the substantial potential of film mulching to enhance root biomass accumulation. Consistent with the trend in root weight, root diameter measurements followed a similar pattern. The average root diameter in the T3 group was 9.97 ± 0.82 mm, a significant 122% increase over the 4.49 ± 0.20 mm of the T1 group. The expansion of the root system not only signifies an increase in crude drug yield but may also be associated with an enhanced capacity for active compound accumulation. These results collectively establish that film mulching is an effective technique for systematically optimizing the agronomic traits of *S. baicalensis*, with the double-layer mulching (T3) mode demonstrating the greatest efficacy in promoting plant height, root biomass accumulation, and root diameter.

### 3.2 Global analysis of transcriptome sequencing data

To elucidate the intrinsic mechanisms by which film mulching regulates the growth and metabolism of *S. baicalensis* at the molecular level, we performed high-throughput transcriptome sequencing on 18 samples from root (TR) and leaf (TL) tissues across the three treatment modalities. The sequencing generated a total of 127.11 Gb of high-quality clean data (detailed in Supplementary Table S2). The mapping rate of clean reads to the reference genome for all samples was within the ideal range of 85.21%–95.56%. Furthermore, the GC content of each library ranged from 45.41% to 47.60%, and the Q30 base percentage was consistently above 94.41%. These quality control metrics confirmed the high quality, sequence uniformity, and low error rate of the transcriptomic data, deeming it suitable for downstream analysis.

**FIGURE 2:**
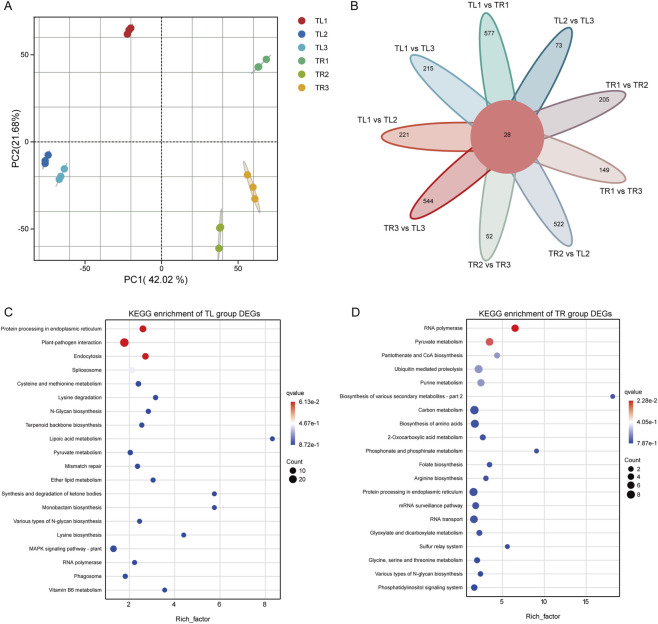
Global analysis of the *S. baicalensis* transcriptome data. **(A)** Principal component analysis based on all gene expression profiles (FPKM values). Different colors and shapes represent samples from different treatments (T1, T2, T3) and tissues (TL: leaf, TR: root). **(B)** Venn diagram showing the number of shared and unique differentially expressed genes (DEGs) among different comparison groups. **(C)** KEGG pathway enrichment bubble chart for DEGs in the leaf (TL) sample group. The size of the bubbles represents the number of enriched genes, and the color intensity corresponds to the enrichment significance (q-value). **(D)** KEGG pathway enrichment bubble chart for DEGs in the root (TR) sample group.

To investigate the overall variation in gene expression profiles among samples and identify the principal drivers of this variation, we first conducted a principal component analysis (PCA) based on the FPKM values of all genes (Figure 2A). The PCA score plot clearly illustrated the sample distribution patterns. The first principal component (PC1), which accounted for 42.02% of the total variance, was primarily responsible for the distinct segregation of all 18 samples into two major clusters corresponding to root (TR) and leaf (TL) tissues, indicating that organ type is the predominant factor determining the gene expression landscape in *S. baicalensis*. The second principal component (PC2) explained 21.68% of the variance and partially distinguished among the different film mulching treatments (T1, T2, T3), particularly within the root tissue samples, which showed a separation trend along the PC2 axis. Notably, all biological replicates clustered tightly together in the PCA space, underscoring the high reproducibility of the experimental design and the reliability of the sequencing data.

Using stringent criteria of a fold change ≥2 and a false discovery rate <0.01, we identified differentially expressed genes (DEGs) across various comparison groups. A Venn diagram visually represents the overlap and uniqueness of these DEGs (Figure 2B). This analysis revealed a core set of 28 DEGs that were common to all defined comparisons, suggesting these genes constitute a fundamental regulatory module responsive to the environmental alterations imposed by the treatments across both tissues.

To gain a deeper understanding of the biological functions executed by the DEGs in different tissues, we performed KEGG pathway enrichment analysis on the DEG sets from leaves (TL) and roots (TR) separately. The results revealed a significant functional differentiation between the two organs (Figures 2C,D). In leaves, DEGs were significantly enriched in pathways such as protein processing in the endoplasmic reticulum, plant-pathogen interaction, spliceosome, and terpenoid backbone biosynthesis. This suggests that the response of leaves, as the primary photosynthetic and defensive organs, to the microenvironmental changes induced by film mulching is mainly focused on maintaining protein homeostasis, activating defense signals, and modulating the synthesis of specific secondary metabolites like terpenoids.

In contrast, DEGs in root tissues exhibited a distinct functional profile. The most significantly enriched pathways included RNA polymerase, pyruvate metabolism, carbon metabolism, biosynthesis of amino acids, and 2-oxocarboxylic acid metabolism. These pathways are central to cellular life, being directly linked to energy production, substance synthesis, and transcriptional regulation. This finding is consistent with our phenotypic observations, indicating that the primary effect of the film mulching treatments is exerted on the roots. This is achieved through a systematic reprogramming of their central carbon and nitrogen metabolism and transcriptional machinery, thereby providing the necessary energy, carbon skeletons, and essential amino acids for rapid root growth and substantial biomass accumulation.

### 3.3 Overall assessment of metabolomic data

To validate whether the systemic changes at the transcriptional level were effectively translated to the metabolite level and to comprehensively map the chemical landscape of *S. baicalensis* under film mulching, we performed broadly targeted metabolomics analysis on the same 18 samples. A total of 2,922 metabolites were accurately identified and quantified across all samples using a UPLC-MS/MS platform operating in multiple reaction monitoring (MRM) mode. These metabolites were annotated and classified into 18 major categories, including flavonoids, amino acids and their derivatives, organic acids, lipids, phenolic acids, and alkaloids.

**FIGURE 3:**
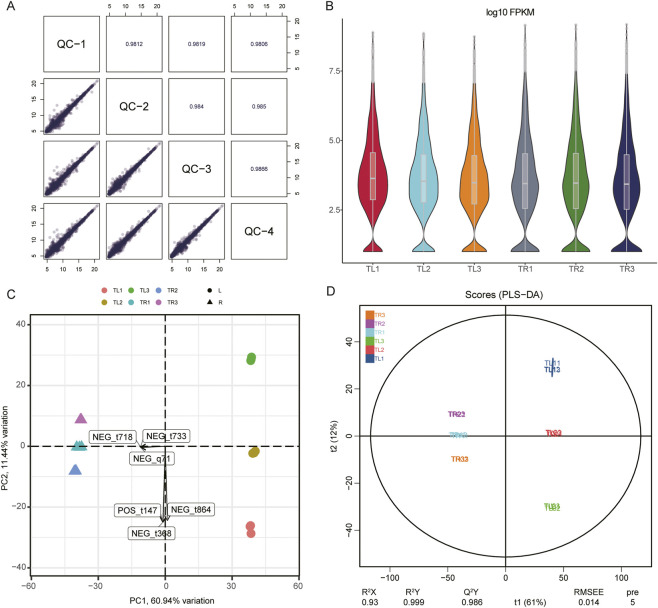
Quality control and global overview of the *S. baicalensis* metabolomic data. **(A)** Correlation matrix heatmap of the quality control (QC) samples, with values representing Pearson correlation coefficients. **(B)** Violin plots of metabolite abundances for all samples, showing the overall data distribution. **(C)** PCA biplot of the metabolomic data. Points represent samples, and arrows represent metabolites that contribute significantly to the sample distribution. **(D)** PLS-DA score plot of the metabolomic data, illustrating the clear separation of sample groups under the supervised model.

The stability and reliability of the experimental procedure were confirmed by assessing the quality control (QC) samples. Pearson correlation coefficients between QC samples across different batches were consistently above 0.98 (Figure 3A), indicating high analytical stability. Violin plots of the overall distribution of metabolite abundances showed similar data distributions and medians across all samples, suggesting the absence of significant systematic bias or batch effects (Figure 3B).

To explore the intrinsic differences in metabolic profiles between treatments and tissues, we first employed unsupervised PCA. The resulting PCA biplot (Figure 3C), consistent with the transcriptomic PCA, showed a clear separation of all samples based on their tissue of origin (root or leaf), reaffirming that tissue type is the principal determinant of chemical composition. Within each tissue cluster, samples from the different mulching treatments also exhibited a clear separation trend.

To further amplify inter-group variance and screen for potential biomarkers, we subsequently applied supervised partial least squares-discriminant analysis (PLS-DA). The PLS-DA score plot demonstrated a more distinct separation between the predefined groups, with all samples forming dense, well-defined clusters (Figure 3D). The model’s performance parameters, with an R2Y of 0.999 and a Q2Y of 0.986, along with validation by a permutation test, confirmed its robustness and ruled out the possibility of overfitting. These results collectively demonstrate that the different film mulching treatments profoundly and systematically influenced the metabolome of *S. baicalensis*, resulting in distinct and discernible metabolic compositions.

### 3.4 WGCNA analysis

**FIGURE 4:**
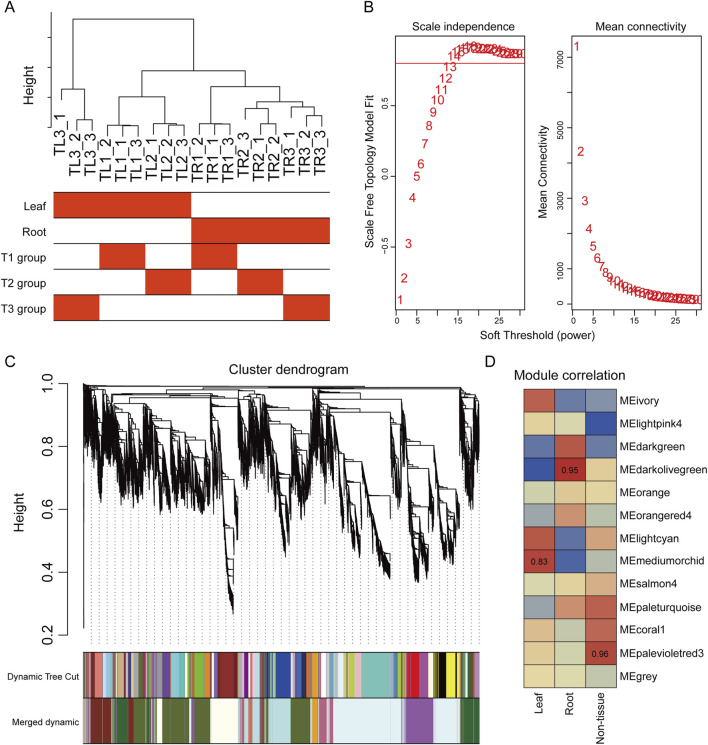
Weighted gene co-expression network analysis (WGCNA) of the *S. baicalensis* transcriptome. **(A)** Sample clustering dendrogram used to check inter-sample correlations and identify outliers. The color bars below represent the corresponding tissue and treatment groups for each sample. **(B)** Analysis for the selection of the soft-thresholding power. The left panel shows the scale independence, and the right panel shows the mean connectivity as a function of the power. The red horizontal line indicates an R-squared threshold of 0.85. **(C)** Gene hierarchical clustering dendrogram. The colored bars below the dendrogram (Dynamic Tree Cut and Merged dynamic) display the 14 co-expression modules identified after dynamic cutting and module merging. **(D)** Module-trait correlation heatmap. Each cell represents the correlation between a module’s eigengene and a specific tissue trait (Leaf, Root). The color intensity signifies the strength of the correlation (red for positive, blue for negative), and the numbers within the cells are the correlation coefficients.

To discern functionally synergistic gene clusters from the complex expression matrix that are tightly associated with tissue specificity and mulching-responsive phenotypes in *S. baicalensis*, we implemented a weighted gene co-expression network analysis (WGCNA) on 16,635 genes exhibiting substantial expression levels and variance. The robustness of the network construction was ensured by first performing a hierarchical clustering of samples to inspect their correlations and exclude potential outliers (Figure 4A).

A soft-thresholding power of 14 was selected as the optimal parameter for achieving a scale-free network topology, as this was the point at which the scale-free topology model fit index (R-squared) first exceeded 0.85 while maintaining high network connectivity (Figure 4B). Based on this, we constructed the gene adjacency matrix and transformed it into a topological overlap matrix (TOM). The dynamic tree cut algorithm identified a total of 14 distinct gene co-expression modules, each designated by a different color (Figure 4C).

To link these abstract gene modules to specific biological questions, we calculated the Pearson correlation coefficients between the module eigengene (ME) of each module and the sample traits (tissue type: leaf, root). The resulting heatmap revealed a strong correlation between module expression patterns and tissue type (Figure 4D). Specifically, the MEorange module exhibited the strongest positive correlation with root tissue, with a correlation coefficient of 0.95 (*p* < 1e-10), while the MEpaleturquoise module showed the highest positive correlation with leaf tissue (r = 0.96, *p* < 1e-10). Additionally, the MElightcyan module was significantly negatively correlated with root tissue (r = −0.83). These highly tissue-specific modules are enriched with gene sets that execute corresponding tissue-specific functions. In particular, the MEorange module, with its high correlation to roots, was identified as a key candidate for containing genes that regulate root growth, biomass accumulation, and the synthesis of medicinal compounds, making it a primary target for elucidating the mechanisms of mulching-induced root promotion.

### 3.5 Analysis of tissue-specific Co-expression networks

**FIGURE 5:**
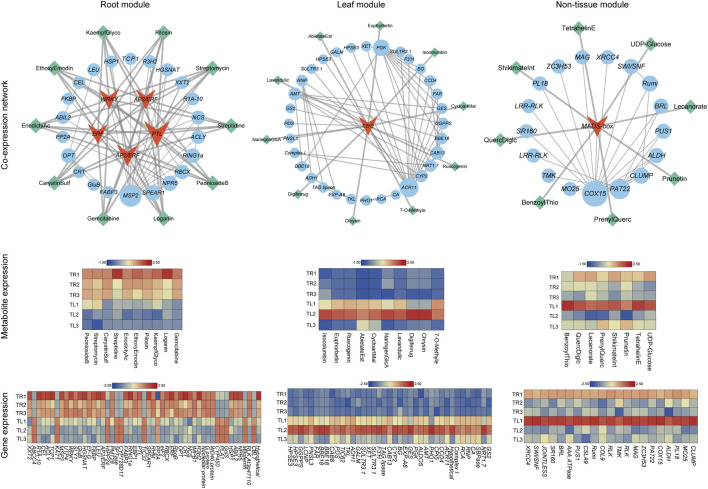
Tissue-specific co-expression networks and the expression patterns of their constituent genes and metabolites. The figure displays co-expression networks for the Root, Leaf, and Non-tissue specific modules. In the networks, red diamond nodes represent core transcription factors (TFs), blue circular nodes represent genes co-expressed with the TFs (weight >0.25), and green diamond nodes represent the top ten metabolites with the strongest Pearson correlation to the core TFs. Node size is proportional to the degree of the gene or metabolite in the network. Below each network is a corresponding heatmap showing the expression levels of all constituent genes and metabolites across the 18 samples. The color scale from blue to red represents low to high expression levels.

Leveraging the core modules identified by WGCNA that are highly coupled with root and leaf functions, we constructed tissue-specific co-expression networks to deeply interrogate the key regulatory factors, particularly transcription factors, and their mediated regulatory pathways (Figure 5).

Within the MEorange module, which is most strongly correlated with root tissue, the co-expression network was centered on core transcription factors. Network topology analysis revealed that multiple members of the AP2/ERF (APETALA2/Ethylene-Responsive Factor) and WRKY transcription factor families occupied hub positions, exhibiting high-connectivity co-expression relationships (weight >0.25) with a large number of downstream genes. These transcription factor families are widely recognized in the plant kingdom for their roles in regulating root development, abiotic stress responses, and the biosynthesis of secondary metabolites. In Lamiaceae plants such as *S. baicalensis*, WRKY transcription factors have been shown to be directly involved in regulating the synthesis of key medicinal compounds like flavonoids, while AP2/ERF factors play crucial roles in responding to environmental factors such as soil moisture and temperature, which are directly influenced by film mulching.

The expression heatmap provided direct evidence supporting this regulatory hypothesis. The core transcription factors and the vast majority of their co-expressed genes within this network exhibited specific and high expression in root samples, with this expression pattern being most pronounced under the T3 treatment. This expression trend was in strong concordance with our phenotypic observation of explosive root biomass growth under the T3 treatment (Figure 1), strongly suggesting that these transcription factors are responders and transducers of the film mulching signal, capable of mediating the initiation of rapid root growth.

In addition to transcription factors, the network also contained numerous downstream genes with well-defined functions. For instance, we identified multiple genes associated with cell wall modification and expansion, such as xyloglucan endotransglucosylase/hydrolases (*XTHs*), as well as cyclins involved in cell division and differentiation. The high expression of these genes in the roots of the T3 treatment provides the molecular basis for the rapid physical expansion of the root system. Concurrently, the network was enriched with various transporter genes, including sugar transporters (*SWEETs*) and amino acid transporters, whose high expression ensures the efficient unloading of photosynthates from the aerial parts to the roots, thereby supplying the raw materials for vigorous metabolic activity.

Crucially, an analysis of the top ten metabolites exhibiting the strongest Pearson correlations with the expression patterns of these core transcription factors revealed an enrichment of several *S. baicalensis*-specific flavonoids, such as baicalin, baicalein, wogonin, and their precursor, naringenin. These flavonoid compounds form the core material basis for the anti-inflammatory, antiviral, and hepatoprotective pharmacological activities of *S. baicalensis*. The significant accumulation of these key medicinal ingredients in the roots of the T3 treatment exhibited high spatiotemporal concordance with the activation of the core transcription factors. This elucidates a sophisticated regulatory mechanism whereby film mulching, by improving the rhizosphere, activates a transcriptional cascade centered on AP2/ERF and WRKY transcription factors. This cascade not only drives growth-related gene programs but also, more importantly, coordinately regulates secondary metabolic flux, channeling a greater proportion of carbon resources toward the biosynthesis of high-value medicinal compounds.

In contrast, the network of the MEpaleturquoise module, which is highly associated with leaf tissue, displayed different functional characteristics. This network was centered on an AP2/ERF family transcription factor, and its co-expressed genes were primarily enriched in pathways related to photosynthesis, photoprotection, and pathogen defense. The strongly correlated metabolites included naringenin and aucubin. Naringenin is a key precursor for the synthesis of UV-absorbing flavonoids, while aucubin is a typical phytochemical defense compound. This indicates that in the leaves, the response network to film mulching is primarily dedicated to optimizing light energy utilization and maintaining defense capabilities against external biotic stress, reflecting its functional specialization as a “source” organ.

The network of the non-tissue-specific module was centered on a MADS-box transcription factor. MADS-box genes are major effectors that regulate plant organogenesis and developmental transitions, such as flowering time. The genes and metabolites in this network exhibited a more basal and ubiquitous expression pattern across different tissues and treatments, suggesting their involvement in the fundamental developmental regulation in response to the mulching treatment, coordinating the growth balance between the aerial and subterranean parts.

### 3.6 Film mulching remodeling the glycolysis/gluconeogenesis pathway

**FIGURE 6:**
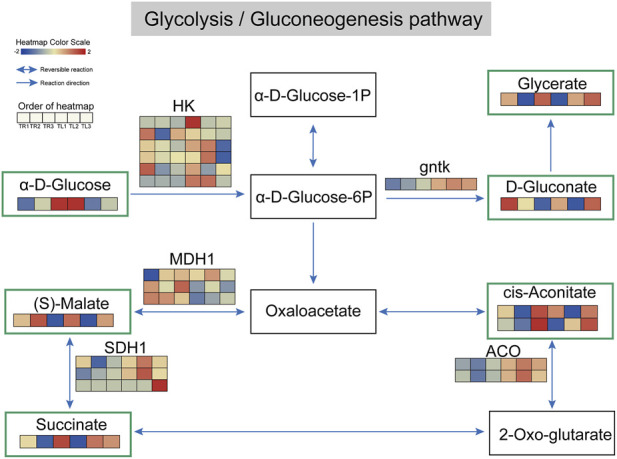
Integrated transcriptomic and metabolomic analysis of the glycolysis/gluconeogenesis pathway. The figure displays key reactions of the glycolysis/gluconeogenesis pathway and the TCA cycle. Squares represent metabolites, with those bordered in green being significantly different metabolites (DEMs) between treatments. Arrows indicate the direction of the reactions. Adjacent to the arrows are the genes encoding the enzymes that catalyze the reactions, with their corresponding expression heatmaps across the 18 samples shown below. The color scale from blue to red represents low to high expression levels.

To further investigate the biochemical basis for the promotion of root growth by film mulching, we performed an integrated transcriptomic and metabolomic analysis of the glycolysis/gluconeogenesis pathway, a central hub of cellular energy metabolism (Figure 6). The analysis revealed that this pathway underwent a systematic and highly tissue-specific metabolic reprogramming in response to the mulching treatments.

In the roots, film mulching, particularly the T3 treatment, significantly activated the entire glycolytic process. At the entry point of the pathway, the concentration of the initial substrate, α-D-glucose, was significantly elevated in the roots of the T2 and T3 treatments, with the T3 group showing a 1.37-fold increase compared to the T1 control. This indicates that the roots, acting as “sink” organs, successfully attracted and accumulated a greater amount of photosynthates from the aerial “source” organs. Concurrently, the expression of the gene encoding hexokinase (*HK*), which catalyzes the first irreversible reaction, was also significantly upregulated, exhibiting a 72.9-fold induction in the T3 roots. Correlation analysis showed that the transcript abundance of *HK* was almost perfectly correlated with *α*-D-glucose content (*r* = 0.987, *p* < 0.05), and the expression of *ACO* was also strongly coupled with *cis*-aconitate levels (*r* = 0.887, *p* < 0.05; Supplementary Table S3). The activation of HK effectively commits a large number of glucose molecules to the metabolic network, thereby substantially increasing the carbon flux into this pathway.

As the metabolic flow proceeded downstream, we observed coordinated changes in multiple key intermediates and enzyme-encoding genes. Pyruvate, the core product of the pathway, is ultimately directed into the tricarboxylic acid (TCA) cycle for complete oxidation. Integrated analysis showed a significant accumulation of several key organic acids of the TCA cycle, including (S)-malate, succinate, and cis-aconitate, in the roots of the T2 and T3 treatments. Simultaneously, the transcript levels of genes encoding key enzymes that catalyze these reactions, such as malate dehydrogenase (*MDH1*) and aconitase (*ACO*), were correspondingly elevated. An active TCA cycle signifies the enhancement of two critical physiological functions: first, it generates a large amount of ATP through oxidative phosphorylation, providing the necessary energy for energy-intensive processes in the roots, such as cell division, expansion, and active transport; second, TCA cycle intermediates, such as oxaloacetate and α-ketoglutarate, serve as essential carbon skeletons for the synthesis of various amino acids, nucleotides, and lipids.

In contrast, the response of the leaves to this pathway was considerably weaker. Although some fluctuations in genes and metabolites were observed, a systematic, pathway-wide upregulation, as seen in the roots, was not evident. This pronounced tissue-specific difference reflects the source-sink relationships and functional division of labor within the plant. By improving the rhizosphere microenvironment, film mulching sends a strong growth signal to the roots, which, in turn, activate the glycolysis/TCA cycle engine to efficiently convert the received carbon resources into the energy and basic building blocks required to drive their physical growth and chemical synthesis.

### 3.7 Film mulching systematically activated root-specific flavonoid biosynthesis pathway

**FIGURE 7:**
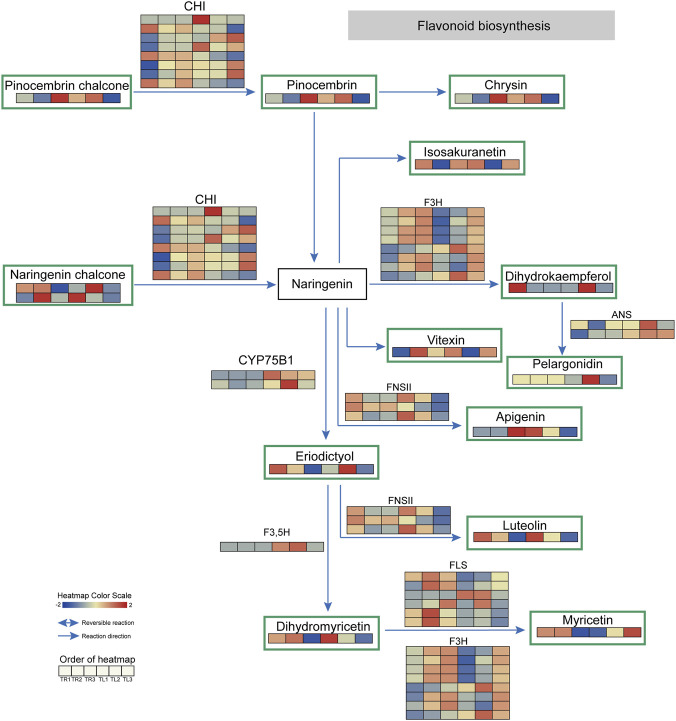
Integrated transcriptomic and metabolomic analysis of the flavonoid biosynthesis pathway. The figure illustrates the major branches of the flavonoid synthesis pathway. Squares represent metabolites, with those bordered in green being differentially expressed metabolites (DEMs). Arrows indicate the direction of the reactions. Adjacent to the squares or arrows are the genes encoding the enzymes that catalyze the relevant reactions, with their corresponding expression heatmaps across the 18 samples shown below. The color scale from blue to red represents low to high expression levels.

Flavonoids are the most crucial and abundant pharmacologically active components in *S. baicalensis*, possessing a wide range of biological activities, including antioxidant and anti-inflammatory effects. Our integrated analysis of the flavonoid biosynthesis pathway revealed a highly root-specific secondary metabolic pathway that is finely regulated by film mulching (Figure 7). The results demonstrate that the T2 and T3 treatments systematically activated the flavonoid synthesis flux in the roots of *S. baicalensis*. From the upstream precursors, naringenin chalcone and the key intermediate naringenin, to various downstream branch products with diverse functions, including apigenin, luteolin, kaempferol, and myricetin, the contents of these metabolites showed a significant and approximately dose-dependent accumulation in the roots of the T2 and T3 treatments. Specifically, the levels of pinocembrin and pinocembrin chalcone in the T3 roots were 2.35-fold and 2.44-fold higher, respectively, than those in the T1 group.

This systematic upregulation at the metabolite level was underpinned by a coordinated activation of genes at the transcriptional level. We found that the expression levels of enzyme-encoding genes that catalyze a series of key steps in this pathway were significantly induced. For instance, the transcript abundances of chalcone isomerase (*CHI*), which catalyzes the cyclization of chalcone to flavanone; flavone synthase II (*FNSII*), which converts flavanone to flavone; and flavanone 3-hydroxylase (*F3H*) and flavonol synthase (*FLS*), which play key roles in the flavonol synthesis pathway, all reached their peak levels in the roots of the T3 treatment (e.g., a 2.02-fold increase in *CHI* expression). Quantitative analysis further confirmed the tight coupling between the transcriptome and metabolome; specifically, the expression of *CHI* was highly correlated with the accumulation of pinocembrin chalcone (*r* = 0.834) and pinocembrin (*r* = 0.821), while *F3H* transcript levels showed a strong correlation with pinocembrin content (*r* = 0.856, *p* = 0.05; Supplementary Table S3). This coordinated, multi-node, and multi-gene upregulation pattern, where the magnitude of gene induction was highly consistent with metabolite enrichment, is characteristic of transcriptional regulation, demonstrating that film mulching can reprogram the entire flavonoid synthesis network by activating upstream regulatory factors.

This finding is consistent with the results from our WGCNA co-expression network analysis. In the root module network, the identified core regulatory factors, including members of the WRKY and AP2/ERF families of transcription factors, have been extensively documented as key transcriptional activators or repressors of the plant flavonoid biosynthesis pathway. The film mulching treatment, acting as an external signal, likely activated these transcription factors in the roots. These transcription factors, in turn, bind to the promoter regions of key enzyme genes in the flavonoid synthesis pathway, systematically enhancing the transcriptional activity of the entire pathway and ultimately leading to the substantial accumulation of various medicinal flavonoid compounds in the roots.

Similar to the glycolysis pathway, the activation of the flavonoid synthesis pathway also exhibited strong root specificity. In the leaves, the overall activity of this pathway was much lower than in the roots and was not sensitive to the mulching treatments. This further substantiates our conclusion that the roots of *S. baicalensis* are the primary sites for the synthesis and accumulation of its medicinal active ingredients. Furthermore, it suggests that the core mechanism by which film mulching enhances the quality of *S. baicalensis* is through its ability to efficiently target and activate the flavonoid secondary metabolism regulatory network in the roots.

### 3.8 qRT-PCR validation of key genes

**FIGURE 8:**
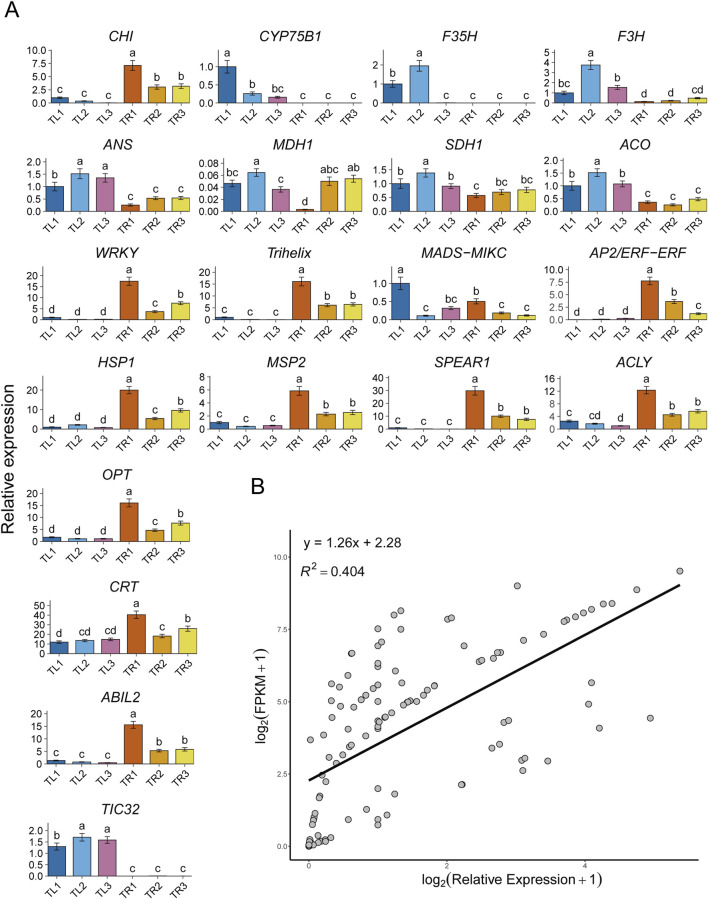
qRT-PCR validation of the transcriptome data. **(A)** Relative expression levels of 20 key genes in different treatment and tissue samples. TL1-3 represent leaf samples, and TR1-3 represent root samples from the different mulching treatments. Data are presented as the mean ± SD. Different lowercase letters above the bars indicate significant differences between groups at the *p* < 0.05 level, as determined by one-way ANOVA followed by an LSD test. **(B)** Linear regression analysis of the qRT-PCR (log2(Relative Expression) + 1) and RNA-seq (log2(FPKM) + 1) expression data for all genes and samples. The linear regression equation and the coefficient of determination (R^2^) are provided.

To further validate the accuracy and reliability of the RNA-seq data, we selected 20 genes that exhibited key regulatory roles or significant expression changes in the multi-omic analyses for validation by quantitative real-time PCR (Figure 8A). These genes included members of important transcription factor families (e.g., WRKY, Trihelix, MADS-MIKC, AP2/ERF-ERF), key enzyme genes of core metabolic pathways (e.g., *CHI*, *ANS*, *MDH1*, *ACO*), and other functional genes that play important roles in the co-expression network (e.g., *HSP1*, *CRT*, *ABIL2*). The expression patterns determined by qRT-PCR were highly consistent with the trends revealed by the RNA-seq data. For example, several transcription factors, such as WRKY, Trihelix, and AP2/ERF-ERF, as well as functional genes like *HSP1*, *MSP2*, *SPEAR1*, and *ACLY*, all showed significantly higher expression levels in the root samples compared to the leaf samples, and their expression peaked in the roots of the T3 treatment. This result strongly confirms the central role of these genes in the root’s response to the film mulching treatment. Conversely, genes such as *CYP75B1* and *TIC32* were predominantly expressed in the leaves, with very low or undetectable expression in the roots, which is also consistent with the transcriptomic data and reflects the tissue specificity of gene expression. Furthermore, the changes in the expression levels of individual genes among the different treatment groups, such as the downward trend of *CHI* expression and the upward trend of *MDH1* expression within the root group, were also validated by qRT-PCR. To quantitatively assess the consistency between the two technologies, we performed a linear regression analysis based on the relative expression levels from qRT-PCR and the FPKM values from RNA-seq for all 20 genes across the 18 samples (Figure 8B). The results showed a significant positive correlation between the qRT-PCR and FPKM expression data after log2(x)+1 transformation. The coefficient of determination (R^2^) of the linear regression model was 0.404, indicating that the quantitative results from the transcriptome were a reasonably good predictor of the qRT-PCR measurements. In summary, the qRT-PCR validation results not only confirmed the expression patterns of the key genes but also demonstrated the overall accuracy and reliability of the transcriptomic data in this study.

## 4 Discussion

### 4.1 Film mulching promotes biomass accumulation in *S. baicalensis*

The significant increase in root biomass of *S. baicalensis* observed in this study is consistent with the well-documented growth-promoting effects of plastic film mulching across diverse cropping systems (Gao et al., 2019). The physiological basis for this macroscopic phenomenon can be attributed to the direct amelioration of soil hydro-thermal conditions. During the early growing season, film mulching effectively elevates the temperature of the arable soil layer, a change that is likely crucial for promoting cell division in the root apical meristem and subsequent cell elongation (González-García et al., 2023). Concurrently, the conservation of soil moisture achieved by suppressing surface evaporation not only mitigates potential drought stress but also creates a favorable medium for the efficient uptake of key mineral nutrients such as nitrogen and phosphorus (Abaza et al., 2023). Our transcriptomic data provide deeper insights into the molecular underpinnings of this process. In the roots of mulched plants, pathways associated with the central carbon metabolism network, particularly glycolysis and the TCA cycle, exhibited systematic transcriptional activation. This suggests that the physically optimized rhizosphere conveys a strong growth signal to the subterranean organs, which in turn respond by upregulating their core energy metabolism. An active TCA cycle is dually significant: it generates substantial ATP through oxidative phosphorylation to energize activities such as root expansion (Alisdair et al., 2004), and its various intermediates, including α-ketoglutarate and oxaloacetate, serve as essential carbon skeletons for the synthesis of fundamental biomolecules like amino acids and nucleotides (Zhang and Fernie, 2018). Furthermore, the observed upregulation of genes encoding sugar transporters, such as *SWEETs*, implies an enhanced efficiency in the allocation of carbohydrates from the photosynthetic “source” tissues to the subterranean “sink” tissues (Chen et al., 2012), thereby ensuring that an ample supply of carbon is efficiently unloaded into the roots to meet the demands of vigorous primary metabolism and structural development.

### 4.2 Film mulching activates root-specific flavonoid biosynthesis

A more compelling finding of this study is that film mulching, while driving biomass growth, did not lead to a dilution of medicinal compounds but instead significantly enhanced the concentration of core flavonoids in the roots of *S. baicalensis*. This result indicates that the effects of the mulching treatment extend beyond a simple fertilization effect, acting instead as an environmental signal that is perceived by the plant and elicits a specific metabolic response. As a major class of plant secondary metabolites, the biosynthesis of flavonoids is often tightly coupled at the molecular level with the plant’s defense response mechanisms to abiotic stress (Nakabayashi et al., 2014). The rhizosphere microenvironment created by film mulching, although generally favorable for growth, may also introduce atypical, mild stress factors, such as elevated soil temperatures or altered local soil gas composition. These signals are likely recognized by the root’s sensory systems, thereby activating endogenous defense-related metabolic programs. It has been established that moderate heat stress can act as an effective elicitor, activating the phenylpropanoid pathway in a variety of plant species (Sharma et al., 2019). Our transcriptomic data revealed a coordinated transcriptional upregulation extending from the key enzyme genes of the upstream phenylpropanoid pathway to the core enzyme genes of the downstream flavonoid-specific synthesis pathway, including chalcone synthase (*CHS*), chalcone isomerase (*CHI*), and flavone synthase (*FNSII*). Exogenous elicitors have been shown to modulate the growth and flavonoid synthesis in *Scutellaria baicalensis* Georgi (Ma J. et al., 2024). In *S. baicalensis* specifically, these genes have been confirmed to be key rate-limiting enzymes in the synthesis of its characteristic flavonoid skeleton (Pei et al., 2022), and their significant upregulation can directly promote the substantial accumulation of landmark medicinal compounds such as baicalin and baicalein.

### 4.3 Multiple transcription factors respond to the mulching signal

Upstream transcription factors often represent the central hub of a metabolic pathway’s response to signals. Through WGCNA, this study successfully identified WRKY and AP2/ERF as the key transcription factor families at the core of the molecular response to film mulching. Crucially, direct experimental evidence in *S. baicalensis* has confirmed that members of these families, such as *SbWRKY75* and *SbWRKY41*, regulate baicalin biosynthesis by directly binding to the promoters of key biosynthetic genes (e.g., *SbCLL-7*, *SbF6H*, and *SbUGT*), as validated by yeast one-hybrid and dual-luciferase assays (Fang et al., 2023). Members of these two families are widely recognized in the plant kingdom as critical nodes for integrating endogenous developmental signals with exogenous environmental stress signals (Phukan et al., 2016). The AP2/ERF family of transcription factors is particularly known for its central regulatory role in response to various abiotic stresses, including temperature, drought, and salinity (Licausi et al., 2013). These factors function as molecular switches that integrate environmental signals—such as the hydro-thermal changes induced by film mulching—into cross-regulatory networks to modulate downstream metabolic flux (Ma Z. et al., 2024). The WRKY family, in turn, has been demonstrated in numerous studies to be a key positive or negative regulator of various plant secondary metabolic pathways, especially those of phenylpropanoid and flavonoid biosynthesis (Schluttenhofer and Yuan, 2015). For instance, in *Arabidopsis* and rice, specific WRKY proteins have been shown to directly bind to the cis-acting elements in the promoters of flavonoid synthesis genes, such as *CHS*, to activate their transcription (Yu et al., 2025). Our integrated network analysis further revealed that within the root-specific co-expression module, the expression of multiple *WRKY* and *AP2/ERF* genes was not only significantly induced by the mulching treatment but was also highly coordinated with that of downstream functional genes, including those involved in cell wall modification (e.g., *XTHs*), central metabolism (e.g., *MDH1*), and flavonoid synthesis (e.g., *CHI*, *FNSII*). This suggests a highly sophisticated transcriptional cascade: the environmental signals triggered by mulching may first be perceived by stress-responsive factors such as AP2/ERF, which then, either directly or indirectly, activate WRKY transcription factors, leading to a cascaded response that systemically reprograms the gene expression network of the roots.

### 4.4 Tissue-specific responses to the mulching treatment

A comparative analysis of the root and leaf tissues revealed a high degree of functional specialization in the response of *S. baicalensis* as an integrated organism to film mulching. Although the physical effects of the mulch are confined to the soil, the resulting physiological effects are indirectly transmitted to the aerial parts through root activity and changes in the plant’s internal source-sink relationships. Our multi-omic data showed that the molecular response of the roots was primarily focused on the activation of central carbon metabolism and flavonoid biosynthesis, which accurately reflects their core biological functions as nutrient-storing “sink” organs and the primary sites for the synthesis and accumulation of medicinal compounds. In stark contrast, the transcriptomic changes in the leaves were significantly enriched in pathways such as protein processing in the endoplasmic reticulum, plant-pathogen interaction, and terpenoid backbone biosynthesis. This differentiated response pattern suggests that the primary strategy of the leaves, as the photosynthetic “source” organs, is to maintain protein homeostasis to ensure the efficient operation of the photosynthetic machinery and to activate basal defense systems to counter potential pathogen threats that may increase due to the altered growth conditions (Agrawal and Fishbein, 2006; Lu and Yao, 2018). This functional division of labor between the aerial and subterranean parts is an adaptive trait for the efficient utilization and allocation of resources. The improved root environment under mulching led to an enhanced metabolic activity in the roots, which transmitted a stronger growth demand signal to the aerial parts. In response, the leaves likely optimized the production of photosynthates and their loading into the phloem to meet the increasing carbohydrate demand of the roots (Ainsworth et al., 2010). Concurrently, a portion of the flavonoids synthesized and accumulated in the roots may also be transported to the aerial parts via the xylem to participate in the construction of a whole-plant systemic defense response (Buer et al., 2010). Thus, film mulching does not act in isolation on the roots but rather initiates a complex, whole-plant response network involving long-distance signaling and resource reallocation.

## 5 Conclusion

This study, through an integrated multi-omic and multi-dimensional analysis, systematically elucidates the molecular mechanisms by which plastic film mulching coordinately promotes both root growth and the accumulation of core medicinal compounds in *Sc. baicalensis*. The research confirms that film mulching, particularly with a double-layer application, significantly enhances root biomass by optimizing rhizosphere hydro-thermal conditions. Evidence at the molecular level indicates that this growth-promoting effect stems from the systematic activation of central carbon metabolism pathways, including glycolysis and the TCA cycle, in the roots, which provides the requisite energy and carbon skeletons for root expansion and structural development. More critically, this study reveals that film mulching acts as an environmental signal that specifically activates the root-centric flavonoid biosynthesis pathway. This process is mediated by a regulatory network centered on the WRKY and AP2/ERF families of transcription factors. These transcription factors are induced in response to the mulching signal and synergistically upregulate a suite of key enzyme genes in the downstream flavonoid synthesis pathway, ultimately leading to the substantial accumulation of landmark active compounds such as baicalin and baicalein in the roots. In summary, plastic film mulching, while driving the primary growth of the medicinal plant *S. baicalensis*, also precisely activates the biosynthetic network of high-value secondary metabolites.

## Data Availability

The datasets presented in this study can be found in online repositories. This data can be found in the NCBI repository under the accession number PRJNA1356960, at https://www.ncbi.nlm.nih.gov/.
